# Analysis of Concentrated COVID-19 Outbreaks in Elderly Facilities in Suita City, Osaka Prefecture, Japan

**DOI:** 10.3390/ijerph20206926

**Published:** 2023-10-15

**Authors:** Toshiyuki Shibata, Sawa Okano, Daisuke Onozuka, Etsuko Ohta, Satoshi Kutsuna

**Affiliations:** 1 Suita City Public Health Center, Suita 564-0072, Japan; toshiyukis@mbox.pref.osaka.lg.jp (T.S.); okano820@city.suita.osaka.jp (S.O.); 2 Department of Infection Prevention and Control, Osaka University Hospital, Suita 565-0871, Japan; onozukad@hp-infect.med.osaka-u.ac.jp (D.O.); ota@hp-infect.med.osaka-u.ac.jp (E.O.)

**Keywords:** COVID-19, nursing homes, outbreak, facility type, incidence rate, positive case rate, elderly

## Abstract

There are several types of facilities for elderly individuals in Japan. Infection control efforts, such as care provision and medical care access, differ according to the type of facility. Elderly individuals at these facilities who were infected with coronavirus disease 2019 (COVID-19) experienced severe illness and mortality. This study aimed to determine the characteristics of concentrated COVID-19 outbreaks that occurred in nursing homes and care facilities in Suita City. During this study, twenty-five elderly facilities in Suita City with a capacity of 40 or more individuals where an outbreak occurred during the sixth or seventh wave of infection were included. We investigated whether there was a difference in the COVID-19 incidence and the percentage of positive cases according to the type of facility. We also investigated the relationship between the facility capacity and positive case rate and that between the number of positive cases and outbreak duration. The incidence rate of COVID-19 was significantly different according to the facility type (*p* < 0.001). No association was found between the facility capacity and positive case rate. The outbreak duration increased as the number of positive cases increased (*p* = 0.004).

## 1. Introduction

The coronavirus disease 2019 (COVID-19) was especially dangerous for elderly individuals, who are more susceptible to severe illness and have higher mortality rates. Nursing homes are places where elderly individuals and others with low resistance to infectious diseases live in groups [1,2]. Since the COVID-19 pandemic first began, it was reported that many deaths could occur in nursing homes [3]. Compared with outsiders, people living in nursing homes were more likely to become infected with COVID-19 [4]. Furthermore, aging was a risk factor for death attributable to COVID-19 [4,5]. The fatality rate in Osaka Prefecture increased with age as follows: less than 0.1% for those in their 50s or younger; 0.33% for those in their 60s; 1.469% for those in their 70s; and 4.45% for those in their 80s or older [6]. Therefore, the nursing home industry experienced a crisis caused by the COVID-19 pandemic [3]. 

After the spread of COVID-19, early reports of outbreaks within care facilities in countries worldwide, including France, Spain, Belgium, Canada, and the United States, were published [7]. Because nursing care facilities are a part of daily life, it is not practical to close them completely, even during the spread of infection; therefore, it is necessary to minimize damage among groups who live there. However, even with thorough infection control measures, it is sometimes difficult to find an appropriate balance between infection control and health conditions at the facility while providing ongoing services [8]. Two studies reported that COVID-19-related deaths among nursing facility residents have accounted for 30–60% [1] and 30–70% [9] of all COVID-19-related deaths in Europe. Similarly, a COVID-19 incidence rate of 51.4% (95% confidence interval [CI]: 35.6–67.0) and a fatality rate of 38.9% (95% CI: 20.3–61.4) were reported among residents in South Korea [10]. Moreover, a mortality rate of approximately 45% was reported for residents of nursing homes in Spain [11].

There have been several reports of relatively low incidence rates of COVID-19 in nursing homes in Japan compared with those in Europe and the United States [12], reporting the impact of COVID-19 on elderly adults living in care facilities compared with those living in the communities [13], the relationship between the number and size of outbreaks at long-term care facilities and morbidity and mortality rates [14], and analyses of factors related to the size of outbreaks at medical and social welfare facilities [15].

Nursing homes in Japan are classified into several types according to the legal long-term care insurance system, and each type has different care provisions and access to medical care. However, we have not found any reports of studies that examined COVID-19 outbreaks according to the types of elderly facilities in Japan. This study investigated the differences in COVID-19 incidence rates and percentage of COVID-19 cases according to the types of nursing home facilities in Suita City to clarify the relationship between the capacity of facilities and positive infection rates and that between outbreak duration and the number of wards at the facility. COVID-19 outbreaks began in nursing homes in Suita City in November 2020. This report describes the characteristics of those outbreaks.

## 2. Methods

There are 105 residential care facilities in Suita City, which is under the jurisdiction of our public health center. During this study, an outbreak was defined as a facility with more than five residents who tested positive for COVID-19, regardless of whether those cases were linked to each other, as in Japan, an outbreak was defined as five or more positive cases [16]. Furthermore, we limited this study to 25 facilities with a capacity of 40 or more individuals that experienced an outbreak during the sixth or seventh wave of infection because the COVID-19 infection rate tended to be higher and more varied among these facilities compared to those with a capacity less than 40 (Supplementary Figure S1). Furthermore, most outbreaks occurred during the sixth and seventh waves of infection (Supplementary Table S1). The characteristics of each facility are shown in Table 1. The onset date of positive cases was considered the date of symptom onset or the test date if asymptomatic. The outbreak duration was considered the number of days from the disease onset date of the first patient to the disease onset date of the last patient. In Japan, the incubation period of the disease in concentrated contacts changed several times during the study period [17,18,19]. Although the incubation period should be considered for COVID-19 convergence, the date and time of the last reported positive case was used as the convergence date to exclude the effect of these changes. When an infection was detected at a facility, the public health center extensively performed repeated polymerase chain reaction (PCR) testing for people at the facility, including those with little contact with infected individuals, to prevent and limit the spread of infection.

A chi-square test was performed to determine if the incidence rate differed according to the facility type. An analysis of variance was performed to determine if the percentage of positive cases differed according to the facility type. Spearman’s rank correlation coefficient was used to determine the relationship between the number of positive cases and outbreak duration. A linear mixed-effects model was used to determine if there was an association between the facility capacity and positive case rate and compare the number of positive cases and outbreak duration. 

## 3. Results

The number of positive cases increased significantly during each successive wave of infection. The predominant variants in Japan during each wave were as follows: the wild-type strain observed during the first wave to the third wave; the alpha variant during the fourth wave; the delta variant during the fifth wave; omicron variants BA.1 and BA.2 during the sixth wave; and the omicron variant BA.5 during the seventh wave. The number of facilities affected during the third, fourth, fifth, sixth, and seventh waves were two, two, two, fourteen, and eleven, respectively (Supplementary Table S1). The study period comprised the sixth and seventh waves when the outbreaks were concentrated.

Table 2 shows the time period of each COVID-19 wave in Osaka Prefecture, the major epidemic strains observed in Osaka Prefecture, the number of positive cases in Suita City, and the number of COVID-19 outbreaks according to the type of nursing home in Suita City from 17 December 2021 until 30 August 2022. The time period of each wave was based on press releases from Osaka Prefecture. Three facilities experienced two outbreaks during different waves. 

Table 3 shows the number of facilities that experienced outbreaks based on the facility type, their ratios, *p*-value according to the chi-square test, and capacity. Long-term care health facilities (85.7%), intensive care homes for the elderly (68.8%), and residential-type fee-based homes for the elderly (50.0%) accounted for more than 50% of the outbreaks. Fee-based homes for the elderly with nursing care (12.5%) and elderly housing with supportive services (7.7%) accounted for less than 50% of the outbreaks. No outbreaks occurred at low-cost homes for the elderly. There was a significant difference in the overall incidence rate according to the facility type (*p* < 0.001).

**Figure 1:**
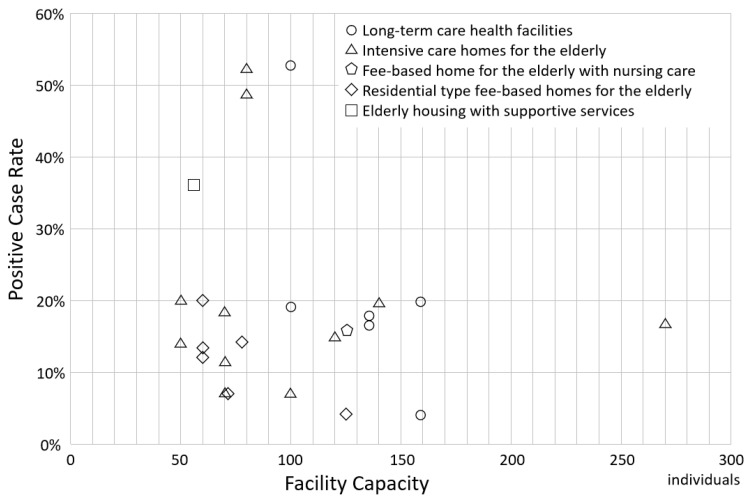
Relationship between facility capacity and positive case rate according to facility type. The x-axis shows the facility capacity, and the y-axis shows the positive case rate.

Figure 1 compares the capacities, outbreaks, and positive case rates of the facilities. The capacity of long-term care health facilities ranged from 100 to 159, and the positive case rate was less than 20%, except for one facility that had a positive case rate of 53.0%. The capacity of intensive care homes for the elderly ranged from 50 to 270, with positive case rates ranging from 7.0% to 52.5%. Residential-type fee-based homes for the elderly had a capacity of 60 to 125 residents, and the positive case rates ranged from 4.0% to 20.0%. The capacity of fee-based homes for the elderly with nursing care was 126, with a positive case rate of 15.9%. The capacity of elderly housing with supportive services was 56, with a positive case rate of 35.7%. All facilities with a capacity of more than 100 had a positive case rate of less than 20%. No significant difference was found in the percentage of positive cases among the five facility types (*p* = 0.486).

**Figure 2:**
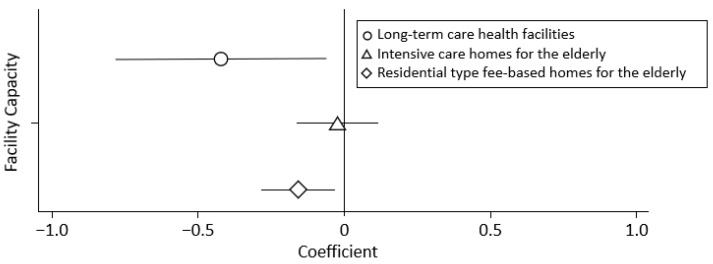
Relationship between the number of residents and positive case rate of long-term care health facilities, intensive care homes for the elderly, and residential type fee-based homes for the elderly. The values are regression coefficients and 95% confidence intervals for each facility.

No association was found between the facility capacity and positive case rate (regression coefficient, −0.038; 95% CI: −0.146 to 0.071; *p* = 0.497). However, associations were found between the facility capacity and positive case rate of long-term care health facilities and residential-type fee-based homes for the elderly, with regression coefficients of −0.422 (95% CI: −0.784 to −0.061; *p* = 0.022) and −0.159 (95% CI: −0.284 to −0.034; *p* = 0.013), respectively (Figure 2).

**Figure 3:**
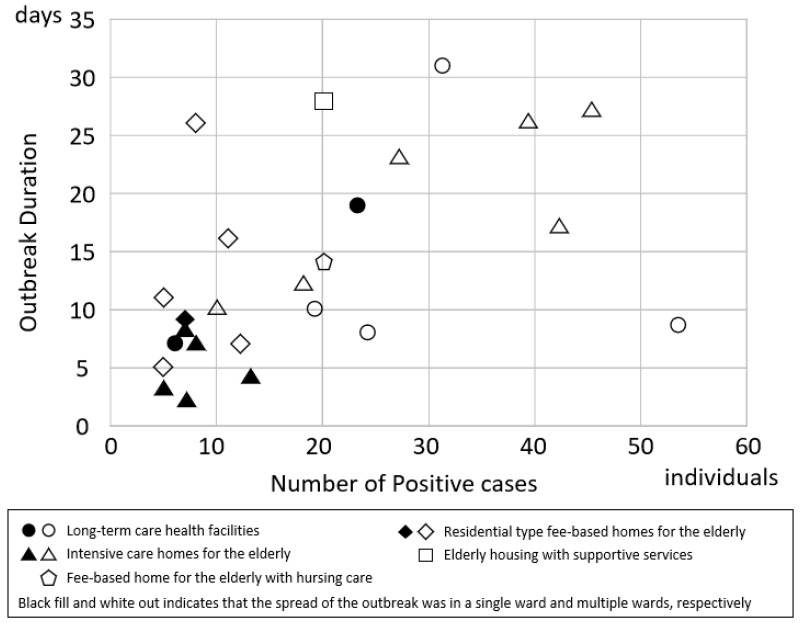
Relationship between the number of positive cases and outbreak duration. The x-axis shows the number of positive cases, and the y-axis shows the outbreak duration.

Figure 3 shows the relationship between the number of positive cases and outbreak duration. As the number of positive cases increased, the outbreak duration tended to increase. Overall, Spearman’s correlation coefficient showed an association between the number of positive cases and outbreak duration (Spearman’s rho = 0.561; *p* = 0.004). Considering the effects of facility capacity, number of positive cases, and facility type, there was a trend toward a shorter outbreak duration if the outbreak occurred in a single ward rather than in multiple wards; however, this was not significant (regression coefficient, −4.08; 95% CI: −10.69 to 2.53; *p* = 0.226).

## 4. Discussion

During the sixth and seventh waves of COVID-19 in Osaka Prefecture, including Suita City, the existing COVID-19 variant was replaced by the highly contagious omicron variant. The sixth and seventh waves accounted for approximately 90% of the total number of new positive cases (as of 30 August 2022). The outbreak rates at nursing homes in the city were similar, with more than 80% of the cases occurring during the sixth and seventh waves. Konetzka et al. reported that large facilities were more likely to experience outbreaks than small facilities [20]. During this study, multiple outbreaks were observed at large facilities. Therefore, it is strongly recommended that infection control measures should be implemented at these facilities.

The rapid spread of COVID-19 within long-term care facilities may have been attributable to a possible delay in the diagnosis because the first cases involved nontypical symptoms [21]. Half of the patients with COVID-19 were asymptomatic pathogen carriers [22], and symptom-based screening alone was insufficient for controlling outbreaks [22,23] because asymptomatic pathogens can be a source of infection. Shi et al. reported that nearly half of the long-term care residents with COVID-19 were asymptomatic at the time of diagnosis [24]. Infection control suggests that early detection minimizes the risk of rapid transmission [10,21]. Danis et al. recommended the early identification of cluster outbreaks and the implementation of testing, infection prevention, infection control, and surveillance programs to reduce the size and severity of outbreaks [1].

The highest outbreak rate (85.7%) occurred in long-term care health facilities; many of these facilities had a relatively large capacity. In the United States, the incidence of COVID-19 was related to the location and size of the facility [25]. Aalto et al. compared the prevalence of COVID-19 and associated mortality of individuals living in nursing homes in 14 countries and observed a significant correlation between the average nursing home size and death attributable to COVID-19 [26]. At the long-term care health facilities and residential-type fee-based homes for the elderly included in this study, the presence of more beds was associated with lower positive case rates, which may be similar to the effect of facility size on outbreaks.

Additionally, because long-term care health facilities were characterized as those that mainly provided rehabilitation and were designed so that residents could return to their own homes, emphasis was placed on rehabilitation care by occupational therapists, physical therapists, and others. Because occupational and physical therapists, rather than caregivers, were often in charge of residents across multiple floors, the number of staff in charge of multiple floors was minimized when outbreaks occurred [27].

Intensive care homes for the elderly had the next highest outbreak rate (68.8%). Outbreaks were considered more likely to occur at intensive care homes for the elderly because residents had more contact with one another, such as when meals were served in the same space, and as the staff provided nursing care to multiple patients. Care services were provided to those who chose them, and some residents were cared for by providers outside the facility. This required attention because of the many points of contact with people outside the facility. For those with 40 or more beds, there was no association between the number of beds and the positive case rate; however, caution was necessary because outbreaks could easily occur, especially at intensive care homes for the elderly. This situation was not considered unique to intensive care homes for the elderly because of the extensive care provided and frequent contact with caregivers.

At some long-term care health facilities and intensive care homes for the elderly with high outbreak rates, resident rooms comprised multiple beds, and it was possible that difficulties in zoning methods contributed to cluster outbreaks; furthermore, densification was an environmental risk factor for COVID-19 [28]. A study performed in Canada also estimated that dense facilities resulted in larger and more fatal COVID-19 outbreaks; for example, it was found that changing a room with four beds to a room with two beds could have averted 19.1% of deaths attributable to COVID-19 [29].

The COVID-19 incidence rates at residential-type fee-based homes for the elderly and fee-based homes for the elderly with nursing care were 27.3% and 22.2%, respectively. It is difficult to explain these incidence rates according to the facility type because the services offered by these facilities vary greatly.

The COVID-19 incidence in elderly housing with supportive services was relatively low (4.3%). Brandén et al. found that the increased mortality rate at nursing homes might have been caused by contact with visitors and caregivers, as well as the poor general health of nursing home residents [30]. Although the residents of elderly housing with supportive services fulfilled both conditions and comprised an environment where it was difficult to ascertain the health status of residents, the low incidence rate may have been attributable to the rare interventions provided by the nursing care staff and the initial healthy status of many residents.

Overall, the facility size had little effect on the percentage of positive cases at facilities with 40 or more beds. However, Dequeker et al. reported that larger nursing homes were at higher risk for long-lasting outbreaks than other nursing homes [31]. The longer outbreak duration and higher number of positive cases in our study may have been similar to those observed by Dequeker et al. [31]. During this study, the overall outbreak duration was longer with a higher number of positive cases, but the outbreak duration varied when the number of positive cases was small. Therefore, the relationship between the number of wards where the outbreak spread and the outbreak duration was examined. When the outbreak spread to multiple wards, the outbreak duration was longer, but not significantly longer. Outbreaks limited to a single ward were considered attributable to the staffing, equipment, zoning, and staff rest area policies. However, one facility experienced a long outbreak duration even though the number of positive cases was small and the infection spread only in a single ward. At that facility, the last positive case developed 9 days after the previous positive case was discovered; this suggests that the longer incubation period of COVID-19 affected the outbreak duration. Conversely, there were many positive cases, but some facilities had relatively short outbreak duration. At one of those facilities, 18 positive cases were identified by screening tests conducted immediately after the first case was identified. At that facility, many of the infected patients were mildly ill or asymptomatic, and the early detection of positive cases was difficult. Most cases involving the omicron strain were mild, and serious illnesses were rare among elderly care facilities in Suita City because the facility residents had received multiple doses of the COVID-19 vaccine by the time of the omicron wave.

Earlier detection of positive cases is necessary to shorten outbreak duration. However, regular testing of asymptomatic cases requires enormous amounts of time and effort. The effectiveness of such testing can be determined by considering the fatality and severity rates of the epidemic strains of COVID-19, as well as the financial and workforce costs.

Elderly individuals experienced increased rates of death because of COVID-19. Ye et al. reported that active discussions of advance care planning increased, and that the number of “do not hospitalize” nursing home residents increased from less than 25% to almost 50% during the COVID-19 pandemic [32]. Additionally, they stated that it is important for all healthcare providers to actively review advance care planning with nursing home residents and their surrogate decision-makers during a pandemic to ensure that care is consistent with their personal goals and avoid unnecessary hospitalizations [32].

This study had some limitations. First, the awareness of infection prevention and the cost of infection control measures differed among employees, even within the same facility type. Administrative inspections conducted by public health centers at facilities where positive cases were detected were not adequately frequent, and the positive cases may not have been fully identified because of labor shortages at the facilities. Second, some physicians at the facility, especially part-time physicians, may not have provided adequate initial treatment for COVID-19. Third, some facilities received guidance regarding infection prevention only from the public health center, whereas other facilities had infection control nurses from hospitals in Suita City as a part of the staff; therefore, access to medical care varied greatly among facilities. Fourth, the number of employees at the facilities could not be considered an influencing factor because the number of part-time employees and employees other than caregivers could not be ascertained. A larger number of employees could have improved the response to infection control measures; however, in some cases, it also could have increased the number of contacts with positive cases. Our public health center survey was not sufficiently detailed to investigate this further. Fifth, it was not possible to examine the impact of the number of residents, the size of the facility, and the number of facility visitors during an outbreak. However, we assumed that the number of residents was usually consistent with the facility capacity because many facilities are almost always fully occupied. We were not able to examine the size of the facilities because the size of private rooms for residents varied, even within the same facility, and shared spaces were utilized in different ways (provision of meals, recreational activities, and temporary suspension of use to prevent infection) depending on the facility. Government policy recommended that visitors should not meet with facility residents; however, because this policy was not legally binding, different facilities handled visitors in different ways.

## 5. Conclusions

The incidence ratio of outbreaks varied by the type of nursing home facility, and associations were found between the capacity of some facility types and positive case rate, but no significant differences in outbreak duration according to the number of wards were observed. Factors other than the type of facility also influenced the impact of COVID-19 outbreaks in nursing homes, and further factor analysis is needed to build future knowledge of outbreak response in nursing homes, considering the characteristics of the facilities.

## Figures and Tables

**Table 1:** Characteristics of facility types.

	Facility Characteristics
Long-term care health facility	Facilities that provide nursing care and functional training under medical supervision, other necessary medical care, and daily life care to individuals requiring long-term care who need support to maintain and restore their physical and mental health functions and to enable them to live at home.
Intensive care home for the elderly	Facilities that provide general nursing care and services such as short-stay medical care and daycare for older adults who need constant nursing care and have difficulty living at home.
Fee-based home for the elderly with nursing care	Nursing care facilities where nursing care staff reside and provide personal care services, such as cleaning and laundry, as well as provide assistance with meals, bathing, and elimination.
Residential-type fee-based home for the elderly	Residential accommodation for older people with services for daily living assistance such as meal provision, cleaning, and laundry.
Elderly housing with supportive services	Barrier-free residences, mainly for older adults who do not require a high level of nursing care, where these elder adults can live with the same degree of freedom as in ordinary residences and still receive services such as safety confirmation and lifestyle consultation.

**Table 2:** The time period of each COVID-19 wave in Osaka Prefecture, the number of positive cases in Suita City, major epidemic strains in Osaka Prefecture, and the number of outbreaks according to the type of nursing home with a capacity of 40 or more individuals in Suita City.

	From	To	The Number of Positive Cases in Suita City	The Major Epidemic Strains	The Number of Outbreaks According to the Type of Nursing Home in Suita City					
					Long-Term Care Health Facility	Intensive Care Home for the Elderly	Fee-Based Home for the Elderly with Nursing Care	Residential-Type Fee-Based Home for the Elderly	Elderly Housing with Supportive Services	Total
Sixth Wave	17 December 2021	24 June 2022	31,156	Omicron variants BA.1 to BA.2	3	6	1	4	0	14
Seventh Wave	25 June 2022	30 August 2022	37,436	Omicron variant BA.5	3	5	0	2	1	11

**Table 3:** Number of facilities that experienced outbreaks based on the facility type and their ratios, *p*-value according to the chi-square test, and capacity.

	The Number of Facilities that Experienced Outbreaks	The Number of Facilities with a Capacity of 40 or More	Ratio	Long-Term Care Health Facility	Intensive Care Home for the Elderly	Fee-Based Home for the Elderly with Nursing Care	Residential-Type Fee-Based Home for the Elderly	Elderly Housing with Supportive Services	Low-Cost Home for the Elderly	The Capacity of Facility that Experienced Outbreaks
Long-term care health facility	6	7	0.857		0.621	0.010	0.173	0.001	0.250	100–159
Intensive care home for the elderly	11	16	0.688	0.621		0.027	0.441	0.002	0.353	50–270
Fee-based home for the elderly with nursing care	1	8	0.125	0.010	0.027		0.158	1.000	1.000	126
Residential-type fee-based home for the elderly	6	12	0.500	0.173	0.441	0.158		0.030	1.000	60–125
Elderly housing with supportive services	1	13	0.077	0.001	0.002	1.000	0.030		1.000	56
Low-cost home for the elderly	0	1	0.000	0.250	0.353	1.000	1.000	1.000		50
Dementia care facility	-	0	-	-	-	-	-	-	-	-

## Data Availability

We are always available to provide the data used in this study as needed.

## Supplementary Materials

The following supporting information can be downloaded at: https://www.mdpi.com/article/10.3390/ijerph20206926/s1, Figure S1: Relationship between facility capacity and positive case rate according to facility type. The x-axis shows the facility capacity, and the y-axis shows the positive case rate; note: we compared the capacities of outbreak facilities in Suita City with their positive case rates. Several facilities with a capacity less than 40 individuals were shown to tend to have higher and more varied positive case rates; Table S1: Time period of each COVID-19 wave in Osaka Prefecture, number of positive cases in Suita City, major epidemic strains in Osaka Prefecture, and number of outbreaks according to the type of nursing home with a capacity of 40 or more individuals in Suita City. 25 out of 31 outbreaks (80.6%) were found in sixth wave or seventh wave.
