# Supplementary material for: Analysis of Concentrated COVID-19 Outbreaks in Elderly Facilities in Suita City, Osaka Prefecture, Japan
Source: Int J Environ Res Public Health. 2023 Oct 15;20(20):6926. doi: 10.3390/ijerph20206926 (PMC10606492; doi:10.3390/ijerph20206926)

Supplementary Materials:

Table S1: Time period of each COVID-19 wave in Osaka Prefecture, number of positive cases in Suita City, major epidemic strains in Osaka Prefecture, and number of outbreaks according to the type of nursing home with a capacity of 40 or more individuals in Suita City. 25 out of 31 outbreaks (80.6%) were found in sixth wave or seventh wave.

	From	To	The number of positive cases in Suita City	The major epidemic strains	The number of outbreaks according to the type of nursing home in Suita City					
					Long-term care health facility	Intensive care home for the elderly	Fee-based home for the elderly with nursing care	Residential type fee-based home for the elderly	Elderly housing with supportive services	Total
First Wave	2020/1/29	2020/6/13	62	Wild-type	0	0	0	0	0	0
Second Wave	2020/6/14	2020/10/9	310	Wild-type	0	0	0	0	0	0
Third Wave	2020/10/10	2021/2/28	1056	Wild-type	0	1	0	1	0	2
Fourth Wave	2021/3/1	2021/6/20	1616	Alpha variant	1	0	0	1	0	2
Fifth Wave	2021/6/21	2021/12/16	3793	Delta variant	0	1	1	0	0	2
Sixth Wave	2021/12/17	2022/6/24	31,156	Omicron variant BA.1 to BA.2	3	6	1	4	0	14
Seventh Wave	2022/6/25	2022/8/30	37,436	Omicron variant BA.5	3	5	0	2	1	11

Figure S1: Relationship between facility capacity and positive case rate according to facility type. The x-axis shows the facility capacity, and the y-axis shows the positive case rate; note: we compared the capacities of outbreak facilities in Suita City with their positive case rates. Several facilities with a capacity less than 40 individuals were shown to tend to have higher and more varied positive case rates.

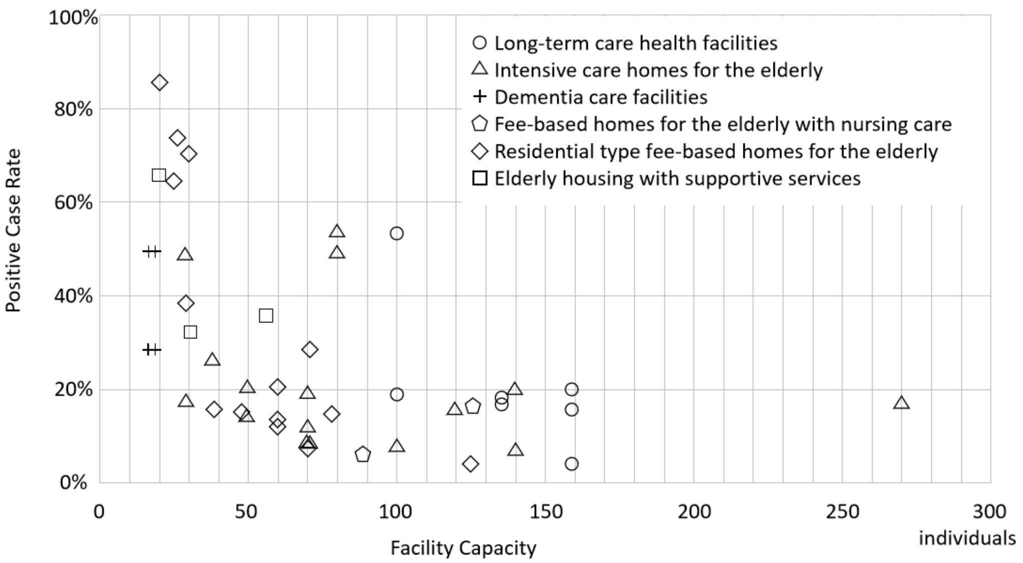

Supplement: Supplementary file 1 [file ijerph-20-06926-s001.zip › ijerph-2587694-supplementary.pdf]
